# Environmental Topology and Water Availability Modulates the Catalytic Activity of β-Galactosidase Entrapped in a Nanosporous Silicate Matrix

**DOI:** 10.1038/srep36593

**Published:** 2016-11-04

**Authors:** M. Ines Burgos, Manuel I. Velasco, Rodolfo H. Acosta, María A. Perillo

**Affiliations:** 1 IIByT-UNC CONICET. Cátedra de Química Biológica, Facultad de Ciencias Exactas Físicas y Naturales, Universidad Nacional de Córdoba. Av. Vélez Sársfield 1611, X5016GCA Córdoba, Argentina; 2 IFEG-UNC CONICET. FaMAF-Universidad Nacional de Córdoba, 5000 Córdoba, Argentina

## Abstract

In the present work we studied the catalytic activity of *E. coli* β-Gal confined in a nanoporous silicate matrix (E_β-Gal_) at different times after the beginning of the sol-gel polymerization process. Enzyme kinetic experiments with two substrates (ONPG and PNPG) that differed in the rate-limiting steps of the reaction mechanism for their β-Gal-catalyzed hydrolysis, measurements of transverse relaxation times (*T*_*2*_) of water protons through ^1^H-NMR, and scanning electron microscopy analysis of the gel nanostructure, were performed. In conjunction, results provided evidence that water availability is crucial for the modulation observed in the catalytic activity of β-Gal as long as water participate in the rate limiting step of the reaction (only with ONPG). In this case, a biphasic rate vs. substrate concentration was obtained exhibiting one phase with catalytic rate constant (*k*_*cA*_), similar to that observed in solution, and another phase with a higher and aging-dependent catalytic rate constant (*k*_*cB*_). More structured water populations (lower *T*_*2*_) correlates with higher catalytic rate constants (*k*_*cB*_). The *T*_*2*_-*k*_*cB*_ negative correlation observed along the aging of gels within the 15-days period assayed reinforces the coupling between water structure and the hydrolysis catalysis inside gels.

The immobilization of proteins by encapsulation in porous glasses through sol-gel techniques is of special interest for the synthesis of biosensors^1^ and as an experimental model for the comprehension of the molecular crowding phenomenon^2^,^3^. In early studies it has been observed that encapsulated proteins preserved most of their native conformation^4^,^5^,^6^ and retained their native spectroscopic properties and certain functional characteristics^5^,^6^,^7^,^8^,^9^,^10^,^11^. However, in most cases encapsulated enzymes suffered a partial loss in their catalytic activity, probably due to the high ethanol content resulting from the polymerization process involved in the sol-gel method. Large molecules are trapped within the porous net while small molecules are able to diffuse throughout the gel. Entrapment of protein molecules in the sol-gel matrix apparently occurs because the silicate polymerizes around the biomolecule and physically traps it in the growing oxide network^12^. It is expected that the macromolecule in this condition would experience a completely different environment from the one found in dilute solutions. Confinement in nanopores would induce diffusional and interfacial effects, local concentration inhomogeneities of the reactants, and changes in the properties of the solvent. Extensive experimental and simulation studies of confined water in nanopores^13^,^14^,^15^,^16^,^17^,^18^,^19^ revealed that water molecules exhibit properties rather different from bulk water. At least two behaviors of water molecules can be discerned inside the pore. One population corresponds to bound water molecules, i.e. molecules strongly adsorbed at a layer close to the surface having a reduced mobility and specific orientations towards the interface. The other population corresponds to free water molecules, i.e. molecules which are less influenced by the pore walls and which exhibit properties close to the bulk values.

β-Galactosidase from Escherichia coli (β-Gal) is a well-studied tetrameric enzyme whose mechanism of hydrolysis was characterized for its natural and artificial substrates^20^,^21^,^22^,^23^. In a previous work we encapsulated β-Gal in a silicate matrix through the sol-gel method and observed improved stability for the encapsulated protein (E_β-Gal_) compared with the same protein in solution (S_β-Gal_)^24^. The present work focuses on the catalytic activity of E_β-Gal_ confined in gels with varied aging times. Differences in the kinetic parameters associated to the hydrolysis of two artificial substrates 2-nitrophenyl-β-D-galactopyranoside (ONPG) and 4-nitrophenyl-β-D-galactopyranoside (PNPG), and transverse relaxation ^1^H-NMR data supported the hypothesis that the structure of water confined inside the nanopores of the silicate matrix would be responsible for the enzymatic activity modulation observed.

## Results

The silicate matrix is usually formed by hydrolysis of an alkoxide precursor followed by condensation to yield a polymeric oxo-bridged SiO network^25^. As long as the system remains wet, the structure and properties of the gel so formed suffers a continuous change through a process known as gel ‘aging’. This occurs through polycondensation and cross-linking reactions taking place in the solid amorphous phase. Thus, spontaneous shrinkage and contraction of the gel networks occurs leading to smaller pores^26^ and this fact may influence on changes in protein activity when confined within it^27^. It is important to note that acidic medium works as a catalyst, inducing an increase in the rate of tetraethyl-orthosilicate (TEOS) hydrolysis and affecting the condensation reactions.

In the present work, enzymatic experiments of E_β-Gal_ aged in contact with aqueous buffer for different periods of time were accompanied with ^1^H-NMR measurements of transverse relaxation times to analyze the molecular mobility of water in the system. Scanning electron microscopic (SEM) images of dried aerogels revealed the morphology of the wet gel with a good approximation but unfortunately, this methodology did not allow us monitor the effect of aging.

### Aerogels morphology

**Figure 1:**
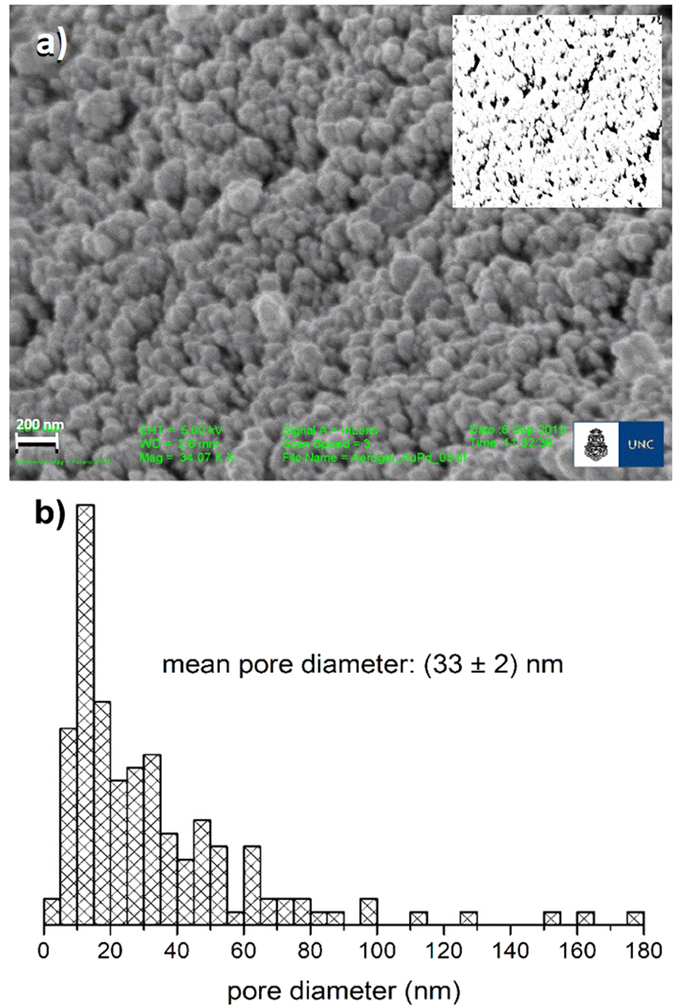
Aerogel morphology. (**a**) SEM image from an aerogel obtained by supercritical drying. Experimental details are described in M&M section. The reference scale bar indicates 200 nm. Inset, equivalent binary image used for the evaluation of porosity. (**b**) Pore diameter distribution in nm units.

For SEM, it is normally required that samples are completely dry, since the specimen chamber is at high vacuum. In turn, the silicate gel encloses a continuous liquid phase in a solid network. Air-drying causes considerable and irreversible contraction of the gel network and induces a flow of liquid from the interior of the body^26^,^28^. At certain point, the capillary forces that arise during the drying process leads to the gel cracking. Therefore, in order to prevent this phenomenon and preserve the structure of the wet samples the silicate gels were dried under supercritical conditions (see the Methods section). The SEM images of aerogel thus obtained, revealed a microscopic structure consistent of agglomerated particles (Fig. 1a). The interstitial spaces between particles (pores) exhibited a wide size distribution within the nanometer scale, between 3 nm and 180 nm diameter, as estimated through the analysis of SEM images (Fig. 1b). Due to resolution limits, pores below 10 nm may have been underestimated however, taking into account that β-Gal diameter is approximately 12–17 nm, smaller pores would not be so relevant for the present work in terms of the environment that pores provide to the enzyme.

### Catalytic activity of β-Gal encapsulated in nanoporous gel

Sol-gel silicate synthesis has been usually performed with a 4:1 H_2_O:TEOS molar ratio which is near the stoichiometric relation of the global reaction. In this condition E_β-Gal_ shows low activity (data not shown) probably due to the fact that ethanol, a secondary product in the hydrolysis of TEOS, is present at high concentrations (>40% V/V) in the reaction media (see Supplementary Fig. S2). It is noteworthy that the catalytic activity of β-Gal decreases up to one third in the presence of 40% V/V ethanol if compared with an ethanol free media (see Supplementary Fig. S1). Previous methods were proposed to prevent ethanol accumulation in presence of the protein^29^. However, we assayed a 20:1 H_2_O:TEOS molar ratio which demonstrated that the amount of ethanol produced by the condensation reaction was around 13% V/V (see Supplementary Fig. S2), a condition that preserves E_β-Gal_ activity.

The experimental set-up allowed the spectrophotometer light beam to cross the gel and the buffer over it and sense simultaneously the concentration of product molecules formed both inside and outside the gel (see Material and Methods and Fig. 5). In these conditions the reaction kinetics became independent of the lag time in the diffusion of the reaction product out of the gel and calculations of E_β-Gal_ activity were simplified compared with our previous work^24^.

**Figure 2:**
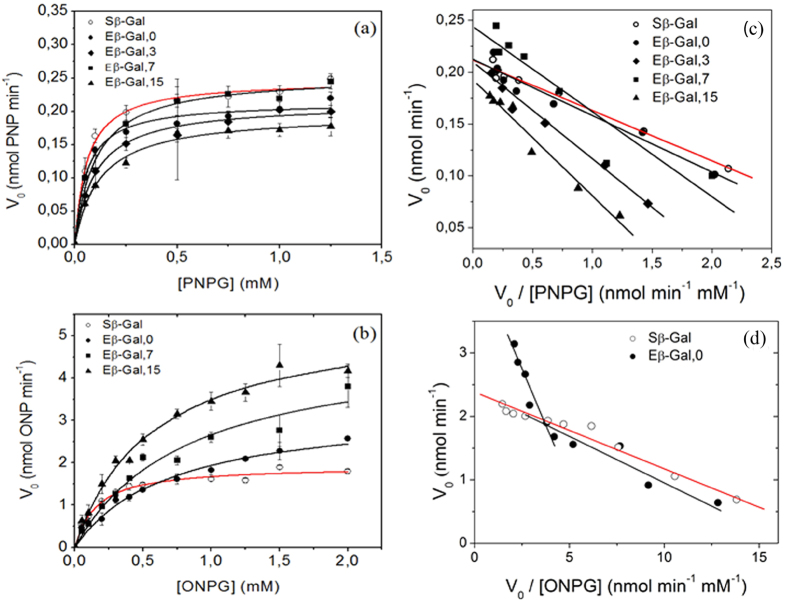
Effect of the substrate type and aging times on the catalytic activity of S_β-Gal_ and E_β-Gal_. Panels (a,b), saturation curves for the hydrolysis of PNPG (**a**) and ONPG (**b**) in buffer solution, in freshly prepared gels (0 days) and in aged gels (3, 7 and 15 days for PNPG and 7 and 15 days for ONPG). Continuous lines represent the fitting of Michaelis-Menten equation (PNPG) or Eq. 1 (ONPG) to experimental data. Panels (c,d), Eadie Hofstee plots for S_β-Gal_ and E_β-Gal_ of PNPG (**c**) and ONPG (**d**) (linearization of curves shown in a and b, respectively). A single straight line in (**c**,**d**) corresponds to the hyperbolic Michaelian behavior in (**a**,**b**). Two straight lines in (**d**) were drawn to highlight the different asymptotic slopes of the non-Michaelian behavior. Red lines correspond to S_β-Gal_. Each point is the average of triplicate determinations in a single experiment and error bars represent the standard deviations. These experiments were repeated at least twice with similar results.

The initial rates of product formation (*V*_*0*_) as a function of substrate concentration were obtained for the two substrate analogues, PNPG and ONPG using soluble enzyme (S_β-Gal_) as well as E_β-Gal_ with different aging times (Fig. 2). Both S_β-Gal_ and E_β-Gal_ exhibited enzymatic activity in all the conditions tested.

A qualitative analysis evidenced that for PNPG the rate of hydrolysis *vs.* substrate concentration curves, obtained with S_β-Gal_ and E_β-Gal_ looked hyperbolic and quite similar to each other (Fig. 2a). Furthermore, the Eadie-Hofstee data analysis of PNPG hydrolysis (Fig. 2c) in both cases (S_β-Gal_ and E_β-Gal_) showed a single straight line supporting a Michaelian behavior for the hydrolysis of this substrate within the whole concentration range studied. Interestingly, the slopes of the linear regression of data for E_β-Gal_ in aged gels were always steeper than those for S_β-Gal_ and for β-Gal in fresh gels (E_β-Gal,0_) which, in turn, were similar to each other.

In the case of ONPG hydrolysis, the initial rate (*V*_*0*_) vs. substrate concentration curves evidenced a qualitatively different behavior between S_β-Gal_ and E_β-Gal_ (Fig. 2b). While the former exhibited a clear hyperbolic shape, the latter deviated from this behavior. Moreover, for substrate concentrations higher than 0.5 mM, the initial rate values for E_β-Gal_ were always higher than those exhibited by S_β-Gal_. At least two slopes were resolved in Eadie-Hofstee plots for ONPG hydrolysis catalyzed by E_β-Gal_ (Fig. 2d). This is in accordance with the deviation from the hyperbolic behavior reflected in the *V*_*0*_ vs. [ONPG] plot (Fig. 2b). Therefore, we hypothesized that each slope would reflect the behavior of different E_β-Gal_ populations. Each population is sensing different environments within the gel. One of them, where β-Gal molecules are confined in large pores, would allow a behavior that resembles that of S_β-Gal_. Other populations of E_β-Gal_ confined in smaller pores, would be those exhibiting higher catalytic activity and would be sensing some effects associated to their proximity to the gel-water interface.

### Kinetic parameters of β-Gal catalyzed PNPG and ONPG hydrolysis

Values of kinetic parameters for S_β-Gal_ catalyzed hydrolysis of PNPG and ONPG (Table 1) were obtained by a non-linear regression analysis of the experimental data shown in Fig. 1, assuming the Michaelis-Menten model. The kinetic parameters obtained with S_β-Gal_ against both substrates were in good agreement with those found in the literature.

### PNPG hydrolysis

A non-linear fit of Michaelis-Menten equation to data from the hydrolysis of PNPG catalyzed with E_β-Gal_ revealed values of *V*_*max*_ similar to those obtained with S_β-Gal_, with no significant effect of the aging time. However, the apparent *K*_*M*_ value with E_β-Gal_ was aging dependent and higher than with S_β-Gal_ (Table 1) coincidently with the steeper slope observed in the Eadie-Hofstee plot (Fig. 2c). It is well known that K_M_ is directly related to the affinity of the enzyme-substrate interaction hence, it is associated to the binding of the substrate to the active site in the enzyme and the availability of substrate. V_max_ is proportional to the amount of active enzyme molecules (or active sites) and *k*_*cat*_, the turnover number, represents the moles of substrate hydrolyzed per mole of enzyme in a unit time. The values of the kinetic parameters determined for PNPG hydrolysis indicate that the main effect of the confinement of β-Gal was probably exerted on the conformation of the active site without a significant effect on the reaction rate constants (thus, on the reaction mechanism).

### ONPG hydrolysis

For the analysis of the ONPG hydrolysis catalyzed by E_β-Gal_, taking in mind the hypothesis of two populations of E_β-Gal_ suggested by the two slopes observed in the Eadie-Hofstee plot (Fig. 2d), we estimated the kinetic parameters assuming that the *V*_*0*_ measured was the resultant of the additive activity of each of the enzyme populations. The initial rate of ONP formation *V*_*0A*_, represented ‘S_β-Gal_ like’ E_β-Gal_ and *V*_*0B*_ corresponded to different species of E_β-Gal_, both of them following a Michaelian kinetics, and contributing to a total *V*_*0*_ according to eq. 1:


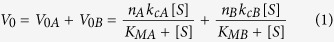


where *n*_*i*_, *k*_*ci*_ and *K*_*Mi*_ are the moles, the catalytic rate constant and the Michaelis constant for each E_β-Gal_ species, A and B, respectively. From the fitting of Eq. 1 to the experimental data, it follows that more than 50% of E_β-Gal_ contributed to the B population term, a proportion which did not vary significantly with aging time. The apparent *K*_*MA*_ values, corresponding to the A population of E_β-Gal_ at 0 days of aging were not statistically different from the *K*_*M*_ for S_β-Gal_ but increased at longer aging times. In turn, the apparent *K*_*MB*_ values obtained were significantly higher than *K*_*MA*_ (Table 1, bold characters). This result suggests that aging affected slightly the apparent affinity of the A component of the enzyme-substrate interaction (compare *K*_*M*_ for ONPG with S_β-Gal_ and *K*_*MA*_ with E_β-Gal_) but induced a strong inhibition of the B component (compare *K*_*M*_ for ONPG with S_β-Gal_ and *K*_*MB*_ with E_β-Gal_) (recall the inverse relationship between K_M_ and affinity, mentioned above). Similarly, while the A component of *V*_*max*_ and *k*_*cat*_ measured with E_β-Gal_ did not differed significantly from the values measured with S_β-Gal_, the B component exhibited *V*_*max*_ and *k*_*cat*_ significantly higher and aging-dependent values. In conjunction these data allow propose the existence of two different population (environments) of E_β-Gal_. Strictly, the apparent *K*_*M*_ values would synthesize the simultaneous effects of several factors including an uneven distribution of ONPG along the porous gel structure due to diffusional restrictions^7^. Additionally, conformational changes suffered by the protein upon encapsulation and different populations of water molecules which are affecting the enzyme-substrate interaction would also contribute to the differential *K*_*M*_values measured. Thus, both E_β-Gal_ populations should not be rationalized merely as different molecular entities but as two kinds of catalytic behaviors. The *k*_*cB*_ values were higher than the catalytic rate constant calculated for S_β-Gal_ and for ‘S_β-Gal_ like’ E_β-Gal_ (*k*_*cA*_) in all the conditions studied (Table 1, bold characters). Moreover, the *k*_*cB*_ increased with the aging time within the time period under consideration (Table 1). These results indicate that there exists a population of E_β-Gal_ associated with a turnover number higher than the value obtained for the soluble enzyme (compare *k*_*cB*_ with the *k*_*cat*_ for S_β-Gal_ in Table 1), and this population suffers the effect of being confined in an environment in continuous evolution (gel aging), while the ‘S_β-Gal_ like’ E_β-Gal_ population might consist of β-Gal molecules entrapped in pores big enough so that the aging effect is sensed with lower intensity.

### Water dynamics in nanoporous gel

The mechanism of β-Gal catalytic activity involves a hydrolysis step (see Fig. 4 below) which may be conditioned by the restricted dynamics of water inside the nanometric pores of the gel. The surface to volume ratio in the gel is high, so most water molecules may experience restricted degrees of freedom and/or they could be in a higher energy state if compared with bulk water^30^. This phenomenon might lead to differential kinetics in processes where water participates in the rate-determining step of the reaction.

**Figure 3:**
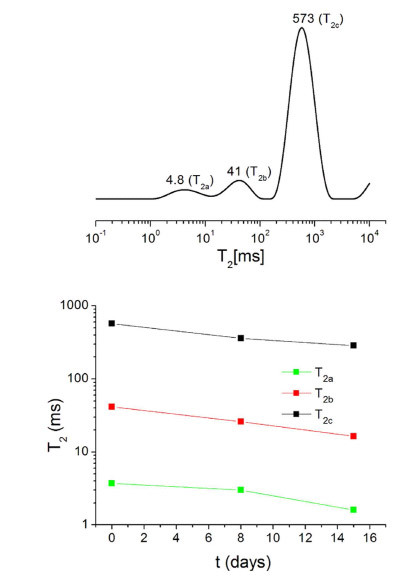
Water dynamics in nanosporous gel. (**a**) Relaxation distribution profile in freshly prepared gels. Three groups of differentially structured water are identified (T_2a_, T_2b_ and T_2c_). (**b**) Mean relaxation times of the individual T_2_ populations as a function of aging time.

Water dynamics in the silicate gel was studied through the transverse relaxation time (*T*_*2*_) of water protons through ^1^H-NMR. It is well known that water exhibits more than one component for *T*_*2*_ in the presence of a hydrophilic silicon interface^19^,^31^. This behavior is usually related to the existence of distinct phases with independent *T*_*2*_ values; where the shortest relaxation times (in the order of a few milliseconds) is associated to water molecules in tight contact with the solid polymeric backbone, while bulk water renders longer relaxation times (in the order of seconds)^32^,^33^,^34^. Additionally, it is expected that in gels a pool of water molecules exhibit an intermediate relaxation behavior (in the order of tens or hundreds of miliseconds), that is, water molecules that are trapped within the gel network or pores, with an intermediate mobility^35^. In the present work *T*_*2*_ values were determined for protons in the silicate gels employing CPMG pulse sequence^36^ and subsequently analyzed with an inverse Laplace transformation. Three *T*_*2*_components (T_2a_, T_2b_ and T_2c_) were obtained indicating that in the sample, water molecules can be categorized in three different mobility pools (Fig. 3a). It is worth to note that due to experimental parameters (see methods section) ^1^H belonging to the silanol groups and water of the buffer do not contribute to the CPMG signal. It must also be recalled that during the experimental time, these three populations may exchange molecules between them, in a dynamic process.

The three *T*_*2*_components (*T*_*2a*_, *T*_*2b*_ and *T*_*2c*_) can be interpreted as water in the first hydration sphere of the silicon polymer (*T*_*2a*;_ 4.8 ms), water within the gel structure (*T*_*2b*_; 40 ms) and water in larger pores or cracks of the gel ball (*T*_*2c*_ ∼ 600 ms). These values are in good agreement with those reported in literature for similar systems^37^. The intensity of the water populations with short *T*_2_ values are small compared to the water contained within the gel, as there is a limit as to the amount of water that can reside in the hydration sphere of the polymer surface.

Along the aging of gel the magnitude of the three *T*_*2*_components decreased (Fig. 3b). We have previously shown that changes in the matrix structure of the gel leads to variations in the *T*_*2*_ values^35^. In this way, the decrease of the relaxation rates can be interpreted as a restriction in the mobility of the water molecules of the different populations due to an increase in the silicate matrix crosslinking.

## Discussion

Sol-gel encapsulation of macromolecules has been proposed as a suitable model system for the study of the effects of crowding and confinement in a living cell^3^.

The gel topology is very sensitive to sol-gel synthesis conditions, for this reason, it is relevant to verify the structure of the gel whenever some of these conditions are changed. In the present work, the sol-gel synthesis was performed, not only at physiological pH, but also at a H_2_O:TEOS molar ratio at which ethanol concentration was low enough to preserve the enzyme catalytic activity. From the SEM images it could be figured that an encapsulated macromolecule could be confined in closed pores or a network of pores interconnected by funnels of different diameters resulting in a heterogeneous environment far from dilute solution conditions. From the pore diameters distribution it was observed that most of them were in the order of β-Gal tetramer dimensions (roughly 17 × 13 × 9 nm)^38^ however, some pores above 100 nm were also found.

**Figure 4:**
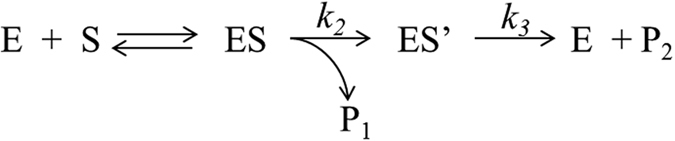
General reaction mechanism of a substrate (S) hydrolysis catalyzed by β-Gal (E). In the present work S represents PNPG or ONPG. ES is the intermediate complex of the substrate bound to the enzyme; P_1_ is the aglycone residue (PNP or ONP); ES’ is an intermediate complex of β-Gal covalently bound to the galactosyl residue; P_2_ in the present work is Galactose, the transfer product resultant from the nucleophilic attack of water (hydrolysis) on ES’.

The activity measured for E_β-Gal_ was similar to that of S_β-Gal_ for the hydrolysis of PNPG and showed higher activity for the hydrolysis of ONPG, which was evidenced by the***k***_***cat***_ values obtained after fitting the curves. For the later, saturation curves were better fitted employing two hyperbolic components (Eq. 1). One of the *k*_*cat*_ values obtained (***k***_***cA***_) was similar to the one obtained with S_β-Gal_ and the other one was significantly higher (***k***_***cB***_). Upon these observations many questions arise. Firstly, why the hydrolysis of ONPG using E_β-Gal_ results biphasic with one of the components having a *k*_*cat*_ higher than that obtained with S_β-Gal_? and why this behavior is not observed when PNPG is employed as substrate? The mechanism of catalysis for β-Gal was proposed by Wallenfels and Malhotra and lately confirmed by Viratelle and co-workers^21^,^22^,^23^,^39^. They postulated two intermediate complexes according to Fig. 4.

The amount of PNP or ONP formed over time and the catalytic rate constant (*k*_*cat*_) can be expressed as shown by Eq. 2 22:


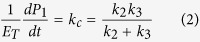


By performing nucleophilic competition experiments with methanol Viratelle and co-workers identified the rate determining step of the reaction (Fig. 4) for many substrates^21^,^22^,^23^. For PNPG the slowest step was the one governed by *k*_*2*_ in which the aglycone was cleaved whereas for ONPG *k*_*2*_and *k*_*3*_ were of the same order of magnitude with *k*_*3*_ ≈ *k*_*2*_/2. This means that the water nucleophilic attack to the galactosyl residue bound to the enzyme is the rate limiting step when OPNG is the substrate but it has no incidence on the kinetics of PNPG hydrolysis. This differential mechanism against both substrates would explain the dissimilar catalytic activity of E_β-Gal_ when compared to S_β-Gal_. For the hydrolysis of PNPG water availability would not be relevant as it does not take part of the rate limiting step. On the contrary, because the hydrolysis step is the slowest for ONPG, it would be expected that changes in water structure and dynamics could influence the net rate in this case.

From *T*_*2*_ measurements it was shown that three different water proton populations could be resolved in the sample. The most abundant was associated with the biggest *T*_*2*_ value (*T*_*2c*_ = 573 ms). Although this correspond to a mobility significantly higher than the other two components (*T*_*2a*_ and *T*_*2b*_) it also reflects certain degree of immobilization considering that water in bulk buffer solution usually renders *T*_*2*_values around 2.5 seconds^30^. This group of water molecules with a dynamics closer to that bulk water would account for the ‘S_β-Gal_ like’ kinetic hydrolysis of ONPG (*k*_*cA*_) by confined enzymes in large pores. The least mobile water molecules are those with the shortest proton relaxation times (*T*_*2a*_) and can be identified as those located in the adsorbed layers closer to the silicate matrix^19^. Between the least mobile water molecules and the bulk type ones there would be a group of water molecules which exhibit intermediate dynamics and that, on average, show the relaxation time *T*_*2b*_. Thus, more structured water, associated with the fastest relaxation times (*T*_*2a*_ and *T*_*2b*_), would be responsible for the higher kinetic constants obtained for the hydrolysis of ONPG (*k*_*cB*_) when data were fitted with Eq. (1).

Moreover, we found an inverse correlation between the enzymatic assays with ONPG and the relaxation experiments as a function of aging time that allow us to propose the following hypothesis. Certain E_β-Gal_ molecules can be confined in big pores (~100 nm) resulting in bulk-like water environment (*T*_*2c*_) and its catalytic parameters resemble those of the free enzyme (*k*_*cA*_ and *K*_*MA*_). Within the environment of nano-sized pores (~10 nm) E_β-Gal_ is in the presence of a higher proportion of water molecules with more restricted degrees of freedom (*T*_*2a*_ and *T*_*2b*_) and it is expected that the kinetic parameters of hydrolysis would differ from those obtained in bulk water (*k*_*cA*_ and *K*_*MA*_). This observation is in agreement with the effect of aging both on water dynamics and on the activity of E_β-Gal_. Since the magnitude of transverse relaxation times of all the resolved water proton populations (*T*_*2a*_, *T*_*2b*_ and *T*_*2c*_) decrease upon gels aging it can be assumed that all the differentially structured water populations on average became less mobile enhancing the catalytic activity of E_β-Gal_ for the hydrolysis of ONPG. Simulation studies of water confined in silica nanopores showed that the first layer of water molecules are expected to be in a specific orientation (named H down orientation)^14^. This fact may account for a more efficient use of water as the substrate in the hydrolysis step to the catalytic reactions.

## Conclusions

In the present work we tried to contribute to the understanding of how the environment within the pore affects the β-Gal catalyzed reaction. It is noteworthy that in bulk, both reactants and catalyst can move freely while in the gel the steric restriction imposed by the pore size to the latter might affect the initial reaction step (enzyme-substrate binding). However, this is not the rate determining step so, it is not surprising that PNPG and ONPG (considering only component A for ONPG) behave in a similar way at least in terms of V_max_ and *k*_*cat*_ values, either in bulk or in the gel. The difference was observed with component B of ONPG, the substrate whose hydrolysis involves a reaction mechanism that uses water in the rate limiting step. This let us arrive to the conclusion that the environment affects the availability of this reactant (water) in terms of dynamics and, possibly, of orientation and is not just a ‘confinement effect’ of the enzyme.

Our results allow us to propose that in this condition of structured water within the nanopores, silica would act as an additional catalyst due to its big surface area, contributing with oriented water molecules to an improved hydrolytic step expressed as a turnover number (*k*_*cB*_) for the E_β-Gal_ catalyzed hydrolysis of ONPG higher than the turnover number obtained with S_β-Gal_. For the hydrolysis of PNPG catalyzed by E_β-Gal_, the *quasi* independence of the global reaction kinetics from the hydrolysis step, can explain the absence of an aging-dependent biphasic kinetics. Finally, our findings highlight the synergism between the confined enzyme in a water structured environment and silica surface in the catalytic properties of nanostructured media. Aging affects K_M_ possibly through a mechanical (decreasing pores size) or osmotic (decreasing free water availability) phenomenon, not explored in the present paper, but which may be related to changes in the enzyme conformation associated to the aging process. Present results, considered in a biological context, would help understanding other systems where polymerization-depolymerization dymamics reproduce situations of environments with wide pore size distribution (e.g. cell cytoplasm)^40^.

## Material and Methods

### Materials

β-galactosidase (β-Gal) from *Escherichia coli* [EC3.2.1.23]. 2-nitrophenyl-β-D-galactopyranoside (ONPG), 4-nitrophenyl-β-D-galactopyranoside (PNPG) and tetraethyl-orthosilicate (TEOS) were purchased from Sigma (St. Louis, MO, USA) and used without further purification.

## Methods

### Silicate Gel Synthesis

Silicate gels were synthesized by the sol-gel method^41^,^42^ modified for protein entrapment by Ellerby *et al*.^5^,^11^ water:TEOS ratio was adjusted to 20:1. Briefly, TEOS (3.0 mL) and deionized water (4.5 mL) were mixed at room temperature, followed by the addition of 0.1 M HCl (0.2 mL) which was used to catalyze the hydrolysis reaction. The reaction mixture was sonicated until it became homogeneous (approximately 4 h) then, equal volumes of the sol and sodium phosphate buffer (0.01 M, pH 6.8) were mixed. Gelation becomes evident within 10 min. For a 100 μL final volume of gel 10 μL of buffer were replaced by the enzyme solution (10 μL, 1 μg. mL^−1^). Samples were stored in polystyrene multi-well plates submerged in buffer, at 4 °C until use. No differences were observed in catalytic activity with or without washing the samples prior to the enzymatic reaction, indicating that enzyme was fully encapsulated. It is important to note that at 20:1 water/TEOS molar ratio the ethanol obtained as secondary product is diluted enough to preserve enzymatic activity (see experiments with the solvatochromic probe merocyanine, as described in the Supplementary Fig. S2).

### Silicate Gel drying and topology analysis

Aeorgels were obtained from gels aged in buffer. For water elimination, samples were firstly soaked in solvents with decreasing polarity according to the following protocol: 5% v/v, pH 7 formaldehyde for 48 h; 30, 50, 70, 80, 90 and 96% v/v ethanol:water solutions for 24 h each one, and 100% ethanol for 12 h. Finally, the samples were soaked in pure acetone prior to CO_2_ replacement and were processed in a critical point dryer (Leica EM CPD030) at the Laboratorio de Microscopía Electrónica y Microanálisis (LABMEM) UNSL-CCT San Luis, San Luis, Argentina. Images of Gold Palladium metalized samples were obtained by SEM (FE-SEM - Carl Zeiss – Sigma operating at 5.00 kV, In-Lens detector) at Lamarx – IFEG (CONICET-Universidad Nacional de Córdoba, Córdoba, Argentina). Pore size distribution was estimated through the analysis of SEM images employing images software analyzer *ImageJ* (Wayne Rasband, NIH, USA). Pore diameters distribution was estimated by analyzing the SEM image shown in Fig. 1 which is representative of a several SEM images. The procedure employing the open access software Image-J is widely used for these tasks (ref. 43 and refs therein). The bar scale provided in the original picture is used to set the pixels scale in nm. The image was thresholded at T = 45. This level corresponds to the grey value between 0 and 255 for which we considered that, compared to the non-thresholded image, all the pores are taken into account at a right size. The resulting binary image was composed of only white and black pixels, with pores represented in black. The pore diameters were measured from this image within the resolution of the technique.

### Enzymatic activity determination

**Figure 5:**
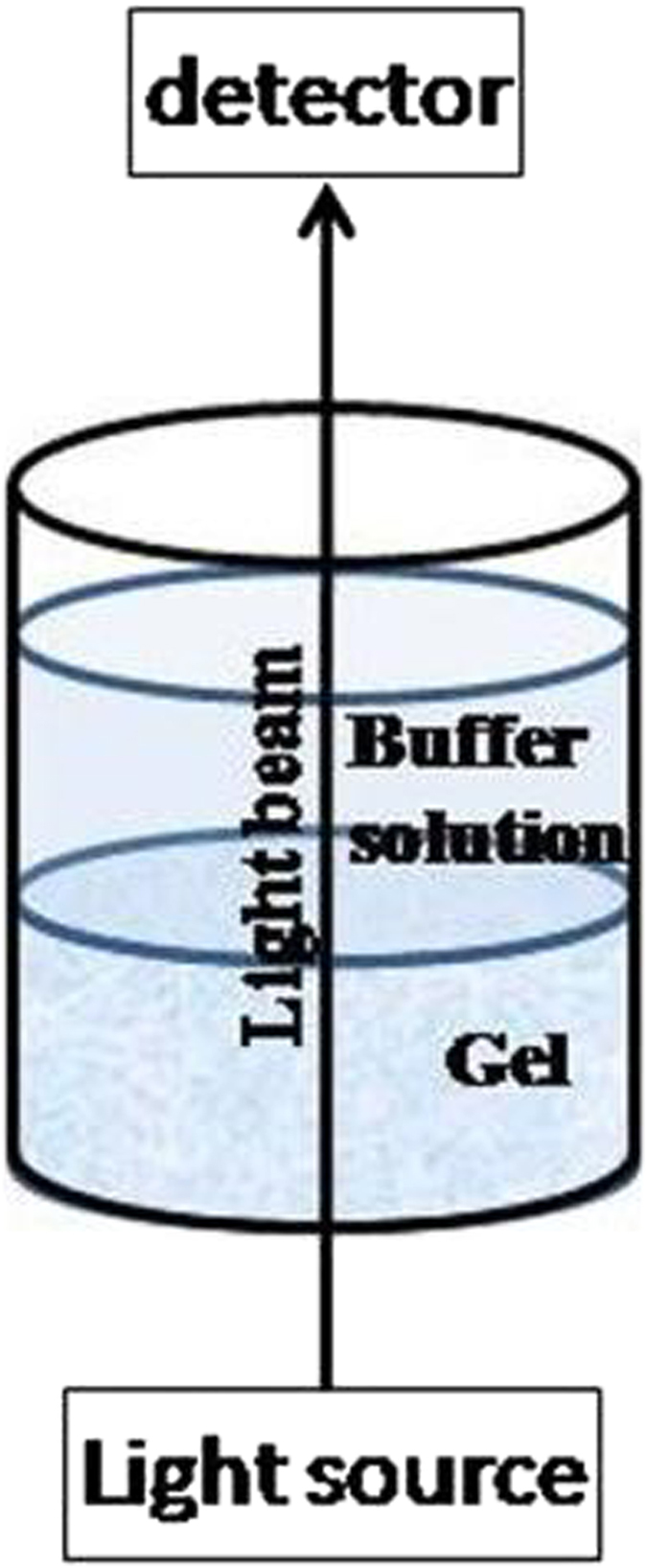
Set up used for spectroscopic measurements.

The hydrolysis reaction catalyzed by β-Gal was studied with each of two substrates, ONPG and PNPG within concentration ranges 0.05 × 10^−3^–2.0 × 10^−3 ^M and 0.05 × 10^−3^–1.25 × 10^−3 ^M, respectively, in 0.10 M Phosphate-buffer, pH 6.8, 10 μL of the substrate solution were incubated at 37 °C in the presence of a silicate gel, with or without (blank) the enzyme entrapped in its interior, and located in a well of a 96 wells-microplate The absorbance of the reaction product was measured at 420 nm (*o*-nitrophenol, ONP) or at 410 nm (*p*-nitrophenol, PNP) in a Multiskan Spectrum (Thermo Fisher Scientific, Finland) (Fig. 5) at 37 °C. This method allowed the measurement of the absorbance of the product inside the gel thus preventing problems on reaction kinetics calculations derived from delays in the diffusion of the product molecule out of the gel^24^.

The molar extinction coefficients for ONP and PNP in buffer (Σ_b,ONP_ = 3969 cm^−1^M^−1^ and Σ_b,PNP_ = 14754 cm^−1^M^−1^) and in the gels (Σ_g,ONP_ = 3902 cm^−1^M^−1^ and Σ_g,PNP_ = 15537 cm^−1^M^−1^) were determined through interpolation in calibration curves (see Supplementary Fig. S3). Calibration curves were also performed in gels aged for 3, 7 and 14 days and no significant differences were observed. In a typical experiment 50 μL of TEOS hydrolyzed (see Enzymatic activity determination above) were mixed with 50 μL of phosphate buffer (100 mM pH 6.8) with or without β-Gal (0.05 μg mL^−1^) at room temperature. After 10 min the gel was formed and 100 μL of buffer with substrate were added. Temperature was set at 37 °C and after 15 min in the case of ONPG and 60 min for PNPG the reaction was stopped with the addition of 30 μL of 14% P/V Na_2_CO_3_. Samples were incubated in dark at room temperature for 30 min in order to achieve a stable value of absorbance before reading. Non- catalytic hydrolysis was discarded with blank measurements. Initial rate (*V*_*0*_) conditions were established by performing product concentration *vs.* time and *V*_*0*_*vs.* β-Gal concentration curves (see Supplementary Fig. S4).

### Data analysis

The values of *K*_*M*_ and *V*_*max*_ were determined by fitting the experimental data from the *V*_*o*_ *vs.* substrate concentration plot to the equation of Michaelis-Menten for PNPG data and to Eq. 1 for OPNG by a computer aided nonlinear regression analysis by the least squares method. For the analysis with Eq. 1 we firstly fit data obtained in buffer and the parameters obtained were employed to feed the fit of E_β-Gal_ data making them fixed. Total protein number (*n*_*T*_ = *n*_*A*_ + *n*_*B*_) is another known value before fit. Therefore, the variable parameters were *n*_*B*_, *K*_*MB*_ and *kc*_*B*_.

### NMR Relaxometry Measurements

Relaxation measurements were performed in a Bruker minispec spectrometer operating at 20 MHz for ^1^H equipped with a BVT3000 sample temperature controller with 0.01 °C stability. Silicate gel spheres were placed individually in a 10 mm tube. A piece of paper soaked in buffer was placed in the upper part of the tube, in order to keep the sample moisture. Transverse proton relaxation times (*T*_*2*_) were measured using a Carr−Purcell−Meiboom−Gill (CPMG) sequence which was applied after a magnetization inversion water suppression pulse. The buffer suppression waiting time was adjusted to 1.5 s. The CPMG parameters were: echo time 0.2 ms, number of echoes 5000, 64 scans and the length of the 90° radiofrequency pulse was 2.45 Ds.

With this echo time it can be assured that the detected signal arises only from water molecules and not from the silicate network, whose ^1^H relaxation times were determined to be on the order of microseconds. The resulting CPMG decay presents a multiple exponential decay, and the T_2_ distribution functions were obtained by using an inverse Laplace transform (ILT) algorithm based on the Tikhonov regularization method provided by Dr. Petrik Galvosas from the Victoria University of Wellington, New Zealand.

All experiments were repeated at least three times for each sample, and a maximum dispersion of 3% in the processed data was obtained.

## Additional Information

**How to cite this article**: Burgos, M. I. *et al*. Environmental Topology and Water Availability Modulates the Catalytic Activity of β-Galactosidase Entrapped in a Nanosporous Silicate Matrix. *Sci. Rep.* **6**, 36593; doi: 10.1038/srep36593 (2016).

**Publisher’s note:** Springer Nature remains neutral with regard to jurisdictional claims in published maps and institutional affiliations.

## Supplementary Material

Supplementary Information

## Figures and Tables

**Table 1:** Apparent kinetic parameters for the hydrolysis of PNPG and ONPG by β-Gal free and encapsulated in fresh and aged gels.

Sample	PNPG			ONPG			
	*V*_*m*_ (nmol min^−1^)	*k*_*c*_‡ (s^−1^)	*K*_*M*_ (mM)	*k*_*cA*_‡ (s^−1^)	*k*_*cB*_‡ (s^−1^)	*K*_*MA*_ (mM)	*K*_*MB*_ (mM)
S_β-Gal_	0.23 ± 0.02*	74 ± 5*	0.059	629 ± 36*	—	0.14 ± 0.01*	—
	0.090^(b)^		0.033^(b)^	750^(b)^		0.11^(b)^	
	0.090^(c)^		0.033^(c)^	800^(c)^		0.11^(c)^	
				600^(a)^		0.1^(a)^	
E_β-Gal (0d)_	0.21 ± 0.01†	68	0.056 ± 0.008	562	1764 ± 142*	0.11	1.4 ± 0.3*
E_β-Gal (3d)_	0.21 ± 0.01†	68	0.10 ± 0.01†	—	—	—	—
E_β-Gal (7d)_	0.25 ± 0.01†	80	0.10 ± 0.01†	587	2648 ± 360*	0.17	1.7 ± 0.4*
E_β-Gal (15d)_	0.20 ± 0.01†	64	0.12 ± 0.01†	617	**3217 ± nd**	0.15	**0.86**

^‡^Calculated for one monomer of a M.W. = 135000.

^*^Values shown represent the mean ± s.e.m. from 2–5 replicates.

^†^Error associated to the fitting procedure.

Parameters were calculated from the non-linear regression analysis of data shown in Fig. 2a,b. Values marked with superscripts (a,b,c) were taken from refs 21, 22, 23.
