# Supplementary material for: Environmental Topology and Water Availability Modulates the Catalytic Activity of β-Galactosidase Entrapped in a Nanosporous Silicate Matrix
Source: Sci Rep. 2016 Nov 4;6:36593. doi: 10.1038/srep36593 (PMC5095660; doi:10.1038/srep36593)
Supplement: Supplementary Information [file srep36593-s1.pdf]

SUPPORTING INFORMATION

Environmental topology and water availability modulates the catalytic activity of β-Galactosidase entrapped in a nanosporous silicate matrix.

M .Ines Burgos[1], Manuel I.Velasco[2], Rodolfo H.Acosta[2], María A. Perillo[1*]

[1]IIByT (CONICET-UNC). Cátedra de Química Biológica, Facultad de Ciencias Exactas Físicas y Naturales, Universidad Nacional de Córdoba. Av. Vélez Sársfield 1611, X5016GCA Córdoba, Argentina. [2]FaMAF-Universidad Nacional de Córdoba and IFEG-CONICET, 5000 Córdoba, Argentina

CONTENTS (S 1 to S 5 refer to page numbers)

S 1 - Title, authors, affiliations and description of supporting information content.

S 2 – Effect of ethanol concentration on β-galactosidase catalytic activity.

S 3 – Determination of ethanol content in silica gels.

S 4 – Calibration curves for the spectrophotometric quantitation of ONP in silica gel.

S 5 – Initial rate conditions determination.

EFFECT OF ETHANOL CONCENTRATION ON β-GALACTOSIDASE CATALYTIC ACTIVITY.

Materials and Methods

All reagents were of analytical grade. The enzyme was incubated in three different conditions (0, 30 and 40 % ethanol in 100 mM phosphate, pH 6.8, buffer) in the presence of the substrate *o*-nitrophenyl galactopyraroside (ONPG) at 37ºC for 15 min. The reaction was stopped with 30 µL of Na_2CO_3 (14% V/V). Two additional samples were pre-incubated for 20 min in the presence of 30 and 40%V/V ethanol, before the addition of ONPG. The absorption spectrum of the reaction product (*o*-nitrophenol, ONP) was obtained in a Spectrophotometer DU 7050 (Beckman).

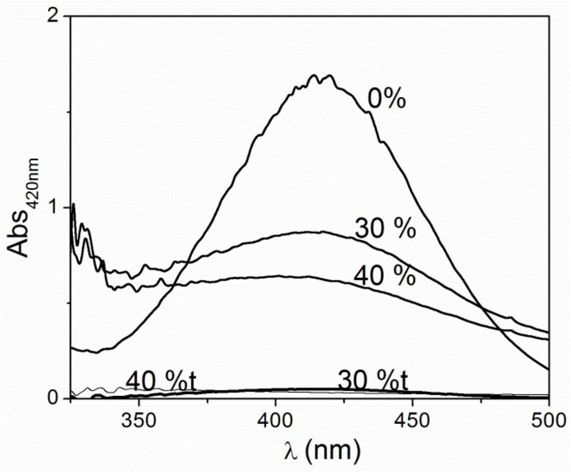

Figure S1. β-Gal activity in ethanol solutions. Absorption spectra of the ONP formed after 15 minutes of incubation in the presence of a constant concentration of β-Gal in 0%v/v, 30% v/v or 40%v/v ethanol solutions. When the enzyme was pre-incubated for 20 min at 25 ºC in the ethylic solutions (30%t and 40%t) the catalytic activity decayed even more. Pre-incubation in the absence of ethanol did not affect the ONP production.

Table S1. ONP formation in the presence of ethanol at different concentrations and for different pre-incubation times

[Ethanol] % (V/V)	*Pre-incubation time (min)*	ε_{420}	A_{420}	*Activity* (µmol min^{-1})
0	0	3969	1.62	27
30	0	5463	0.81	10
30	20	5463	0.07	0.9
40	0	5851	0.57	6.5
40	20	5851	0.02	0.3

ε_{420}, extinction coefficient at 420 nm; A_{420}, absorbance at 420 nm.

DETERMINATION OF ETHANOL CONTENT IN SILICATE GELS

Materials and Methods

Merocyanine 540 (Sigma) samples were a 1/50 dilution from the stock solution prepared in ethanol (analytical grade), in different ethanol/buffer mixtures (0%, 20%, 30%, 40%, 50% and 100%V/V). Silicate gels (SG) were prepared as described in the main manuscript with the addition of 2 mL of Merocyanine 540 stock solution before the start of the condensation reaction. Absorption spectra were recorded in a Multiskan Spectrum (Thermo Fisher Scientific, Finland). The longest wavelength peak (λ_P) for each spectrum was determined using a graphic software (Origin 7).

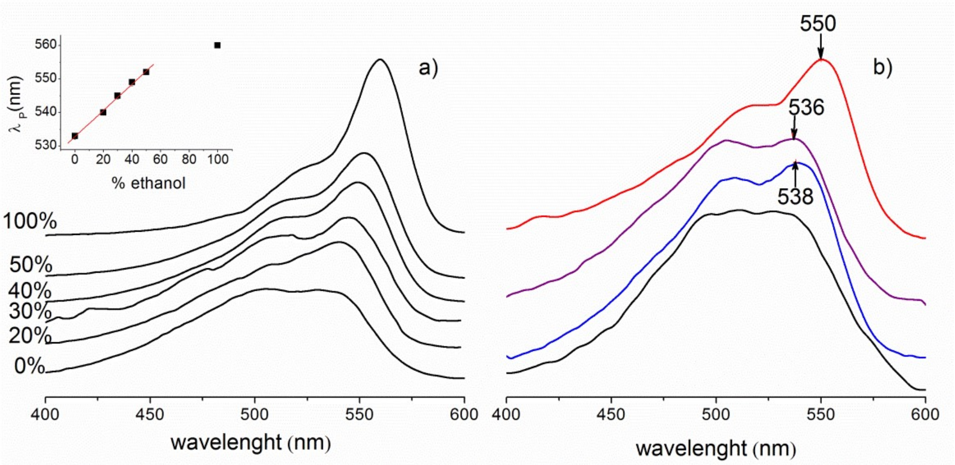

Figure S2. Ethanol content determination in silicate gels (SG).

a) Absorbance spectra of Merocyanine 540 in aqueous solutions with the percent ethanol contents indicated by the numbers at the left. The peak at the longest wavelength (λ_P) experienced a bathochromic shift as the percentage of ethanol was raised. A straight line was fitted to the λ_P vs. [ethanol] plot (inset) within the 0-50% [ethanol] range by applying a linear regression analysis ($\lambda_P = 532.8 + 0.39$ [%ethanol]; r = 0.99).

b) Absorbance spectra of Merocyanine 540 in water (—) and inside SG synthesized from different H_2O:TEOS molar ratios and incubation times after the synthesis: i) SG 20:1, 15 min. (—); ii) SG 20:1-60 min. (—); iii) SG 4:1-15 min. (—). Arrows point to the λ_P value in each case. Ethanol content in SGs is a product of the polymerization reaction in each condition. It was estimated by interpolating the λ_P from spectra in panel b, in the regression line. Results obtained were 13%v/v, 8% v/v and 44%v/v, for samples i, ii and iii, respectively.

CALIBRATION CURVES FOR THE SPECTROPHOTOMETRIC CUANTITATION OF ONP IN SILICA GEL

Materials and Methods

ONP and other reagents were of analytical grade. ONP solutions of different concentrations were prepared in buffer phosphate (pH 6.8, 100 mM) and in SG samples (see M&M from the main manuscript) embedded in buffer. Absorbance was measured at 420 nm for ONP in buffer and in SG samples freshly prepared and aged for 3, 7 and 14 days in the presence of a known amount of ONP. Data within the linear range, in each case, were submitted to linear regression analysis.

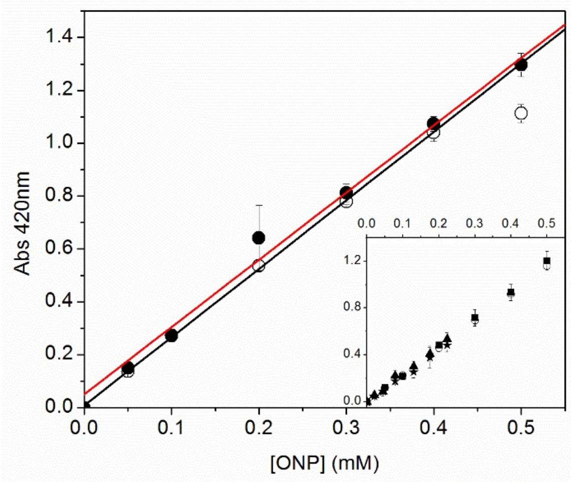

Figure S3. Calibration curves for ONP inside SG. Linear behavior was obtained up to 0.4 mM for ONP in solution (open symbols) and up to 0.5 mM for ONP inside freshly prepared gels (filled symbols). The molar absorptivity coefficients (ε_{420}) were calculated through linear regression of the data and similar values were obtained from both curves (black line for ONP solution and red line for encapsulated ONP). The linear response of ONP was stable along time inside the gel (see inset): (■) 3 days, (▲) 7 days, (★) 14 days and the same values of ε_{420} were calculated in these conditions.

INITIAL RATE CONDITIONS DETERMINATION

Materials and Methods

ONPG and PNPG were purchased from Sigma (St. Louis, MO, USA). All other reagents were of analytical grade. ONPG and PNPG were dissolved in buffer at the desired concentrations and added to the enzyme in solution or encapsulated in wet gels. These experiments were performed in cell culture multi-wells. The absorbance at 420 nm or 410 nm was measured in order to determine the number of micromoles of ONP or PNP, respectively, produced at each point of time.

a) **b)**

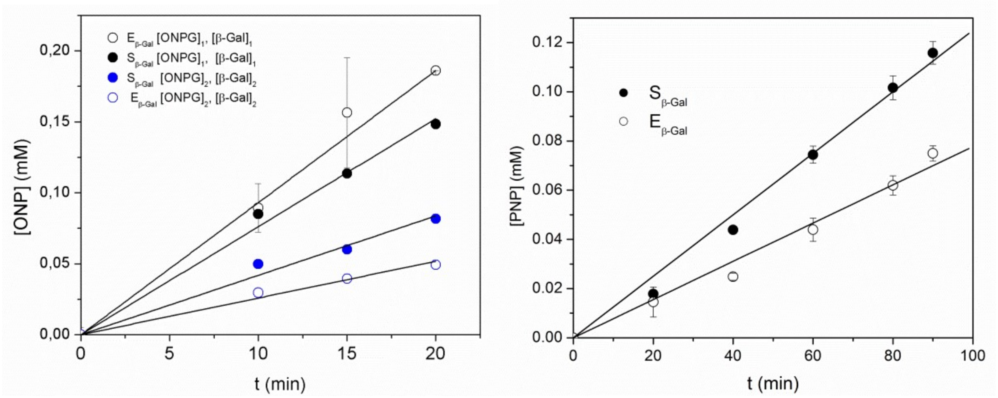

Figure S4. Product concentration vs. time plots.

Hydrolysis of ONPG (a) or PNPG (b). catalyzed by free ($S_{\beta\text{-Gal}}$) and encapsulated ($E_{\beta\text{-Gal}}$) β-Gal. In (a) ONPG at 1.2 mM (●, ○) or 0.05 mM (●, ○) concentrations and in the presence of β-Gal (0.033 μg/mL (●, ○) and 0.066 μg/mL (●, ○) concentrations). In (b) the substrate PNPG (0.5mM) was assayed in the presence of 0.05 μg/mL $S_{\beta\text{-Gal}}$ (●) or $E_{\beta\text{-Gal}}$ (○).
